# Undifferentiated pleomorphic sarcoma of bone (UPSB) treated in the German-speaking countries. A report of 132 unselected patients from the Cooperative Osteosarcoma Study Group (COSS)

**DOI:** 10.1007/s00432-025-06405-5

**Published:** 2026-01-20

**Authors:** Stefan S. Bielack, Dimosthenis Andreou, Daniel Baumhoer, Claudia Blattmann, Godehard Friedel, Birgit Fröhlich, Semi Ben Harrabi, Leo Kager, Thekla von Kalle, Torsten Kessler, Matthias Kevric, Antonia Knoll, Thomas Kühne, Peter Reichardt, Benjamin Sorg, Reinhard Windhager, Stefanie Hecker-Nolting

**Affiliations:** 1 Klinikum Stuttgart – Olgahospital, Stuttgart Cancer Center, Zentrum für Kinder-, Jugend- und Frauenmedizin, Pädiatrie 5 (Onkologie, Hämatologie, Immunologie), Kriegsbergstr. 62, 70174 Stuttgart, Germany; 2 Pädiatrische Hämatologie Und Onkologie, Klinik für Kinder- und Jugendmedizin, Universitätsklinikum Münster, Münster, Germany; 3 Institut für Interdisziplinäre Sarkomtherapie und Forschung, Klinik für Tumororthopädie und Sarkomchirurgie, Zentrum für Orthopädie und Unfallchirurgie, Universitätsklinikum Essen, Essen, Germany; 4 Knochentumor-Referenzzentrum, Institut für medizinische Genetik und Pathologie, Universitätsspital Basel, Basel, Switzerland; 5 Basel Research Centre for Child Health, Basel, Switzerland; 6 Universitätsklinik für Thorax-, Herz- und Gefäßchirurgie - Sektion Thoraxchirurgie, Universitätsklinikum Tübingen, Tübingen, Germany; 7 Klinik für Radioonkologie und Strahlentherapie, Radiologische Klinik, Universitätsklinikum Heidelberg, Heidelberg, Germany; 8 St. Anna Kinderspital, Universitätsklinik für Kinder- und Jugendheilkunde der Medizinischen Universität Wien, Vienna, Austria; 9 St. Anna Children’s Cancer Research Institute (CCRI), Vienna, Austria; 10 Radiologisches Institut (Kinderradiologie), Zentrum für Kinder-, Jugend- und Frauenmedizin, Klinikum Stuttgart – Olgahospital, Stuttgart, Germany; 11 Medizinische Klinik A – Hämatologie, Hämostaseologie, Onkologie und Pneumonologie, Universitätsklinikum Münster, Münster, Germany; 12 Abteilung für Pädiatrische Onkologie/Hämatologie, Universitäts-Kinderspital beider Basel, Basel, Switzerland; 13 Klinik für Onkologie und Palliativmedizin, Helios Klinikum Berlin-Buch, Berlin, Germany; 14 Universitätsklinik für Orthopädie und Unfallchirurgie, Klinische Abteilung für Orthopädie, Medizinische Universität Wien, Vienna, Austria

**Keywords:** Undifferentiated pleomorphic sarcoma of bone, Chemotherapy, Surgery, Radiotherapy, Prognosis

## Abstract

**Purpose:**

To describe Undifferentiated Pleomorphic Sarcoma of Bone (UPSB) treated in Germany, Austria, or Switzerland and using the same treatment-approach as for osteosarcoma.

**Patients and methods:**

The database of the Cooperative Osteosarcoma Study Group (COSS) was screened for UPSB. Eligible patients were evaluated for patient, tumor, and treatment related variables, and outcomes.

**Results:**

One-hundred thirty-two eligible patients were identified (median age 41.7 (range: 10.3–74.6) years; males 58%; preceding malignancies 11%; tumor-sites extremities 86%, trunk 12%, head & neck 2%). Relative tumor size was < 1/3 of the involved bone in 83% of evaluable cases, pathologic fractures were present in 14% (limb-primaries only), and primary distant metastases in 6%. All patients received chemotherapy and 96% surgery for their primary (81% limb-salvage for extremity lesions). The response rate to preoperative chemotherapy was 38%. After a median follow-up of 3.9 (1 day–34.1) years for event-free and 5.2 (0.2–34.1) years for overall survival, the 5 year event-free and overall survival probabilities were 63% (standard error: 5%) and 70% (4%), respectively. Younger patient age, an extremity tumor, and localized disease predicted superior outcomes, pathologic fractures and limb-salvage surgery worse. The extent of tumor response to pre-operative chemotherapy seemed to have no impact on prognosis.

**Discussion:**

UPSB generally affects considerably older persons than does the more frequent osteosarcoma. Despite a lower response rate to preoperative chemotherapy, its prognosis is at least comparable, if not better. Several prognostic factors impacting outcome could be defined. These results provide a benchmark for this rare disease.

## Introduction

Undifferentiated Pleomorphic Sarcoma of Bone (UPSB), formerly named Malignant Fibrous Histiocytoma of Bone (MFHB), represents a rare intraosseous malignancy. Morphologically, this tumor closely resembles osteosarcoma but lacks osteoid formation. UPSB must be distinguished from Undifferentiated Pleomorphic Sarcoma of the Soft Tissues, the latter being one of the more frequent soft-tissue sarcomas of the adult population and treated accordingly (WHO Classification of Tumours 2020).

If left without systemic therapy, UPSB, like osteosarcoma, carries a considerable risk of metastatic spread. Based upon histologic and clinical similarities to this more common malignancy of bone, it is usually treated according to principles established there (Strauss et al. 2021; Pediatric Treatment Editorial and Board 2020). Surgery and multi-agent chemotherapy represent the mainstay of treatment. The background upon which decision making is based on is, however, limited. Only few series have gathered more than fifty affected patients. These were generally retrospective (Capanna et al. 1984; Nishida et al. 1997; Bielack et al. 1999; Nagano et al. 2021; Boudou-Rouquette et al. 2022; Gusho et al. 2022). UPSB was the focus of only very few prospective series in highly selected groups of patients (Bramwell et al. 1999; Palmerini et al. 2023).

We set out to further clinically delineate this tumor and its prognosis. The Cooperative Osteosarcoma Study Group’s (COSS) database offered a unique opportunity to do so. The large, multi-centric COSS-group covers the three German speaking countries of Europe. It has been registering UPSB alongside osteosarcoma from its advent over 45 years ago. It was always recommended to treat UPSB according to the same principles as osteosarcoma. This paper now reports upon the group’s experience with this rare osseous malignancy.

## Patients and methods

### Patient selection and data collection

COSS includes centers from Germany, Austria, and Switzerland and registers patients with osteosarcomas and related malignancies. Its database was now searched for patients with UPSB diagnosed 1980–2020. They were included into this analysis unless their follow-up was < 100 days without having suffered an event. Patients were followed until 2021 or the date of last available information, respectively.

All COSS-study and -registry protocols were performed in accordance with the Code of Ethics of the World Medical Association (Declaration of Helsinki) and approved by the appropriate ethics committee (Ethik-Kommission bei der Ärztekammer Hamburg nos. 500, 1147; Ethikkommission der Ärztekammer Westfalen-Lippe und der Westfälischen-Wilhelms Universität nos. 182/98 Biel2, 4IV Bie 2, 4 I Bielack, 5 V. Bielack; and Ethik-Kommission an der Medizinischen Fakultät der Eberhard-Karls-Universität und am Universitätsklinikum Tübingen no. 5 V Bielack). Informed consent was required from all patients and/or their legal guardians, whichever appropriate.

COSS-recruitment policies as well as the treatment of enrolled patients, which include those with UPSB, have been described in detail (Bielack et al. 2009, 2023a; Ferrari et al. 2018; Smeland et al. 2019). Demographic and therapy data were collected prospectively. Additional information was collected retrospectively from available status report forms, radiology, pathology, and surgery reports, as well as progress letters. All patient-charts at the central COSS-office were screened for additional relevant information by the first author.

The diagnosis of UPSB required histologic verification, preferentially with reference-pathology. Local and systemic staging procedures were performed according to standards defined for osteosarcoma. These included investigations of the primary tumor, the lung, and distant bones. X-rays of the primary and the lungs as well as bone-scintigraphy were always prescribed. The use of other investigations (computed tomography and/or magnetic resonance imaging and/or positron emission tomography) varied with time and availability.

Systemic treatment of UPSB, including pre- and postoperative chemotherapy, followed the guidelines and guidance developed for osteosarcoma. Drugs administered almost always included doxorubicin, high-dose methotrexate (with limitations according to age in patients over 40 years), and cisplatin. Ifosfamide was prescribed in a considerable number of regimens. Other agents were rarely part of the protocols. Local therapy was by complete surgical removal of the primary as well as any primary metastases, if present. The goal of surgical therapy was to achieve wide surgical margins (Enneking et al. 1980). The histologic response of the primary to pre-operative chemotherapy was graded according to Salzer-Kuntschik et al (1983). Radiotherapy was not part of any COSS-regimen but to be considered in inoperable situations. The COSS-office together with panels of experts was available for guidance in case of questions regarding local or systemic therapies or treatment-complications.

The date of diagnostic biopsy was used as the starting date for all survival-analyses. Follow-up was until last available information or death, whichever appropriate. Event-free survival was calculated until the date of recurrence, date of last contact, or date of death, whichever first, overall-survival until death or last contact, respectively. Patients not having achieved a macroscopically complete remission considering all sites of disease were assumed to have suffered an event on day 1. The sites involved by first recurrences were coded. Secondary malignancies were counted but not considered events.

Survival-analyses were performed by the Kaplan–Meier method with 95%-confidence estimates (Kaplan and Meier 1958). Comparisons were made using the log-rank test (Mantel 1966). *P *values < 0.05 were considered significant, no correction for multiple testing was made. Multivariate Cox-models were calculated for extremity tumors. They included those variables which were associated with *p *values < 0.05 upon univariate testing and for which information was available for > 95% of patients. Statistical analyses were carried out using the SPSS statistical software packet (IBM Corp. Released 2022. IBM SPSS Statistics for Windows, SPSS version Version 29.0.0.0, Armonk, NY: IBM Corp.).

## Results

### Patients

A search of the database detected 150 tumors registered as UPSB. Of these, 6 had to be excluded because of later changes in histological classification (3 osteosarcomas, 1 synovial sarcoma with t(X;18), 1 PPM1F-FOXO1 positive surface-sarcoma, 1 mesenchymal chondrosarcoma with HEY1-NCOA2 fusion). Five tumors turned out to be undifferentiated pleomorphic sarcomas of soft-tissues upon chart-review. A further 2 UPSB were only registered upon relapse and 5 had a follow-up of < 100 days without suffering an event.

This left 132 individuals, 77 (58%) males and 55 (42%) females, from Germany (113), Austria (14), or Switzerland (5), registered by pediatric oncologists in 24 (18%) cases and by others in 108 (72%).

A genetic tumor-predisposition was reported for 2 (2%) patients (1 each familial retinoblastoma, Peutz-Jeghers syndrome).

Malignancies preceding UPSB had occurred in 15 (11%) patients. These were diagnosed a median of 11.6 (range: 1.7–36.7) years prior (1 no data) and had been 12 solid tumors (5 carcinomas (2 uterus, 1 breast, 1 bladder, 1 palate), 2 sarcomas (1 Ewing, 1 myxoid liposarcoma), 2 germ-cell tumors (1 ovary, 1 testis), 1 medulloblastoma, 1 melanoma, 1 retinoblastoma) and 3 hematologic malignancies (2 B-cell non-Hodgkin-, 1 Hodgkin-lymphoma). Their therapy had been by surgery in 9, radiotherapy in 12, and/or chemotherapy in 8 cases.

All UPSB were graded as high-grade tumors. COSS-reference pathology was available for 82 (62%) tumors.

The median age at which UPSB was diagnosed was 41.7 (10.3–74.6) years. Twenty-four (18%) patients were in their 2nd decade of life, 18 (14%) their 3rd, 17 (13%) their 4th, 29 (22%) their 5th, 32 (25%) their 6th, 10 (8%) their 7th, and 2 (2%) in their 8th.

The median duration of symptoms prior to diagnostic surgery, reported for 92 individuals, was 95 (1–701) days.

Primary tumor-sites were extremities in 114 (86%; femur 81 (proximal 15, diaphysis 5, distal 61), tibia 24 (proximal 20, diaphysis 3, distal 1), humerus 6 (proximal 5, distal 1), distal radius 1, distal ulna 1, foot 1), the axial skeleton in 16 (12%; pelvis 11, sacrum 1, lumbar spine 1, scapula 2, clavicle 1), and the head & neck in 2 (2%; 1 mandible, 1 os petrosum).

Among extremity tumors, size in relation to the affected bone was reported for 71 UPSB, < 1/3 in 59 (83%) of these. Sixteen (14%) of 114 extremity tumors were complicated by pathologic fractures which arose prior to chemotherapy in 15/16 (94%).

Primary distant metastases were reported for 8 (6%) patients (lungs 5, osseous 5, others 2; multiple mentions possible).

All 132 patients received chemotherapy (no surgery 5, preoperative only 10, postoperative only 29, both 87; unknown 1) for a median of 220 (1–444) days (unknown 2). Drugs administered were reported for 127 individuals and included doxorubicin (124 (98%)), cisplatin (118 (93%)), ifosfamide (104 (82%)), high-dose methotrexate (81 (64%)), and others (16 (13%)).

As for local therapy, 127(96%) primaries proceeded to surgery. In the 112/114 (98%) operated extremity tumors, the type of surgery was known for 110 (limb-salvage 89 (81%), amputation 17 (15%), rotation-plasty 4 (4%)). In those 97 tumors operated upon only after pre-operative chemotherapy, information on tumor-response was available for 79. Less than 10% of the primary tumor was found viable in 30 (38%) of these patients.

Local radiotherapy at a median dose of 52 (2–66) Gray was documented for 11 cases (8%; 3 without, 8 with surgery (of these 5 prior to chemotherapy)).

As a result of therapy, 121 (92%) patients achieved a macroscopically complete remission. Local tumor tissue plus/minus metastases were still present in 8, only metastases in 3 individuals.

Median follow-up for event-free survival was 3.9 (1 day–34.1) years. During this period, 8 patients developed unrelated malignancies, not counted as events (1 had also suffered a malignancy, medulloblastoma, prior to UPSB, another suffered fom Peutz-Jeghers syndrome). These secondary malignancies developed 6.9 (0.5–29.4) years after UPS when patients were 46.2 (13.6–65.4) years old. There were 3 AML/MDS, 4 carcinomas (2 breast, 1 prostate, 1 jejunum), and 1 chondrosarcoma.

First events occurred in 54 patients and were 11 failures to achieve remission (see above), 33 UPS-recurrences (28 metastatic (lung 21, bone 4, brain 2, liver 2, spleen 1, pancreas 1, distant soft-tissues 1; multiple mentions possible), 3 local, 2 combined (both local recurrence and simultaneous lung metastases), 4 deaths of other causes (2 secondary AML, 1 car-accident, 1 pancreatitis), and 6 deaths of unknown causes. Seventy-eight patients remained event-free, their median follow-up was 6.4 (0.3–34.1) years.

Median follow-up for overall survival was 5.2 (0.2–34.1) years for all 132 patients and 6.6 (0.3–34.1) years for 87 survivors. Forty-five individuals died after a median of 3.0 (0.2–31.1) years. Those 33 patients reported as dying from UPSB did so 2.5 (0.2–16.4) years after diagnosis (9 without ever having achieved a remission, 24 recurrences (20 1st, 2 2nd, 2 3rd)). Five patients died from other (4 as first event plus 1 therapy-related death at recurrence) and 7 from unknown causes (6 in 1st UPSB-remission, 1 at 1st recurrence).

**Fig. 1:**
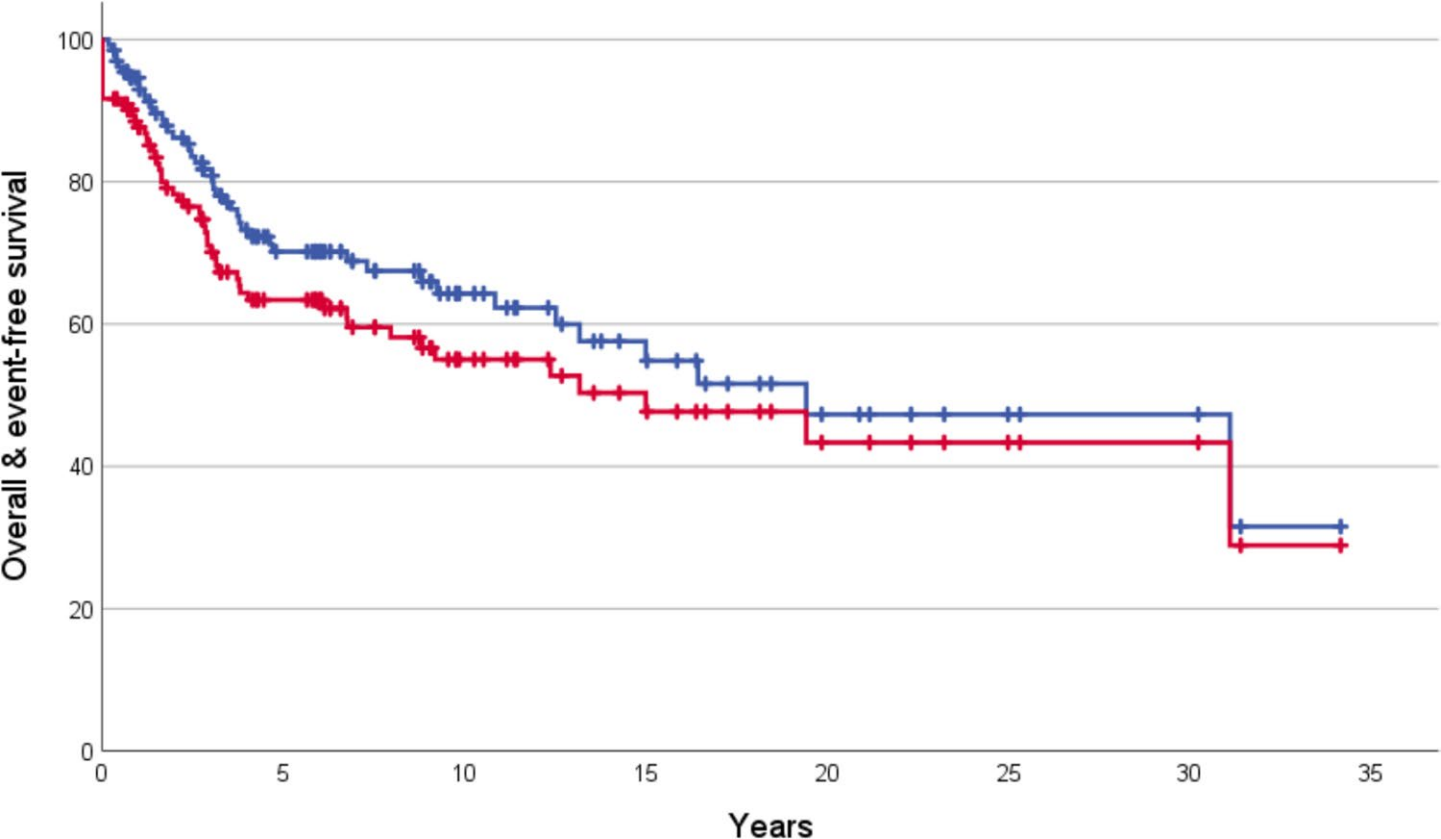
Overall (blue) and event-free survival of 132 individuals suffering from UPSB

The overall-survival probabilities at 1, 2, 5, and 10 years were 95% (standard error: 2%), 86% (3%), 70% (4%), and 64% (5%) for all 132 patients. The corresponding probabilities for event-free survival were 88% (3%), 78% (4%), 63% (5%), and 55% (5%) (Fig. 1). The following prognostic factors were found to be significantly negative for overall and event-free survival at p < 0.01 for the cohort of all patients: Age ≥ 40 years at diagnosis, primary tumor-site outside of the extremities, and primary metastases. Failure to achieve a macroscopically complete surgical remission was significantly negative for overall-survival (event-free not eligible). Further details by tumor-site are presented in Table 1. Multivariate Cox models for event-free and overall survival are shown in Table 2.

**Table 1:** Outcome of patients with OUPS five years after diagnosis, given for the total cohort of 132 patients (left) and divided by primary tumor-sites (limb center, others right)

	All patients					Extremity primaries					Trunk or head & neck primaries				
	n	OS	p	EFS	P	n	OS	p	EFS	p	n	OS	p	EFS	p
All patients	132	70 (4)	–	63 (5)	–	114	76 (4)	–	68 (5)	–	18	36 (12)	–	40 (12)	–
Pathology confirmed by a named COSS-pathologist															
Present	82	66 (6)	0.075	60 (6)	0.114	69	74 (6)	0.331	66 (6)	0.378	13	28 (13)	0.375	30 (13)	0.137
Absent	50	77 (7)		69 /7)		45	80 (7)		70 (8)		5	60 (22)		60 (22)	
Age at diagnosis															
< 40 years	59	84 (5)	< 0.001	75 (6)	0.003	56	85 (5)	0.002	75 (6)	0.014	3	^a^	0.368	^a^	0.680
≥ 40 years	73	59 (6)		54 (6)		58	67 (7)		60 (7)		15	29 (13)		32 (12)	
Gender															
Female	55	73 (7)	0.109	68 (7)	0.218	47	78 (7)	0.127	71 (7)	0.238	8	47 (19)	0.337	50 (18)	0.391
Male	77	68 (6)		60 (6)		67	75 &6)		65 (6)		10	25 (15)		30 (15)	
Symptom duration															
< 90 days	45	69 (8)	0.699	70 (7)	0.142	37	77 (8)	0.413	78 (6)	0.040	8	33 (18)	0.742	38 (17)	0.581
≥ 90 days	47	70 (7)		54 (8)		41	74 (8)		55 (9)		6	44 (22)		50 (20)	
*Not specified*	40					36					4				
Tumor site															
Extremity	114	76 (4)	< 0.001	68 (5)	. < 0.001	114	76 (4)	–	68 (5)	–			–		
Non-extremity	18	36 (12)		38 (12)							18	36 (12)		38 (12)	
Tumor size < 1/3 of the involved bone															
Yes	59	72 (6)	0.181	67 (6)	0.778	59	72 (6)	0.181	67 (6)	0.778					
No	12	89 (11)		57 (17)		12	89 (11)		57 (17)						
*No data or non-extremity*	43					43					18				
Pathological fracture (extremities only)															
None	98	76 (5)	0.981	71 (5)	0.032	98	76 (5)	0.981	71 (5)	0.032					
Present	16	78 (11)		49 (14)		16	78 (11)		49 (14)						
*Not eligible*	18					–					18				
Primary metastases															
Absent	124	74 (4)	< 0.001	67 (5)	< 0.001	108	80 (4)	< 0.001	71 (5)	< 0.001	16	41 (13)	0.114	43 (13)	0.076
Present	8	0 (0)		not reached		6	0 (0)		not reached		2	^a^			
Type of surgery (extremity tumors only)															
Ablative	21	93 (6)	0.024	75 (11)	0.366	21	93 (6)	0.024	75 (11)	0.366	21				
Limb-salvage	89	74 (5)		68 (5)		89	74 (5)		68 (5)		89				
*Not specified*	…0.2					…0.2									
*No surgery*	2					2									
Tumor-response to preoperative chemotherapy															
Good	30	75 (9)	0.465	74 (9)	0.496	25	80 (9)	0.331	78 (9)	0.409	5	40 (30)	0.956	53 (25)	0.720
*Grade 1*	12	79 (14)		79 (14)		9	83 (15)		83 (15)		3	a			
*Grade 2*	7	80 (18)		83 (15)		6	80 (18)		80 (18)		1	a			
*Grade 3*	11	72 (14)		64 (15)		10	79 (13)		70 (15)		1	a			
Poor	49	77 (6)		73 (7)		45	79 (7)		75 (7)		4	^a^			
*Grade 4*	23	86 (8)		77 (9)		21	89 (7)		79 (9)		2	^a^			
*Grade 5*	24	70 (10)		71 (10)		22	72 (11)		73 (10)		2	^a^			
*Grade 6*	2	^b^		^b^		2	^b^		^b^		0	^a^			
*No surgery, no preop. chemo, or no data*	53					44					9				
Complete macroscopic surgical remission of all disease-sites															
Achieved	121	76 (4)	< 0.001	69 (5)	^a^	108	79 (4)	< 0.001	^b^		13	50 (15)	< 0.001	^b^	
Not achieved	11	9 (9)				6	17 (15)				5	0 (0)			

^a^not analyzed as less than 5 patients

^b^not eligible to be analyzed as absence to achieve remission was counted as an event by definition

**Table 2:** Multivariate Cox models of overall and event-free survival for 110 patients with extremity osteosarcomas and information regarding all tested variables

	Overall survival		Event-free survival	
Variable	Relative risk	*p*	Relative risk	*p*
Age ≥ 40 years	2.482	0.032	2.086	0.045
Primary metastases	2.356	0.215	3.626	0.029
Pathologic fracture	1.286	0.658	1.798	0.152
Limb-salvage surgery	3.602	0.085	1.304	0.559
No complete remission	24.677	< 0.001	^a^	

^a^not eligible for testing as failure to achieve remission constitutes an event by definition

## Discussion

This report covers four decades of experience with UPSB gathered in the three German-speaking countries of Europe. To our knowledge, it represents one of the largest cohorts of such tumors studied so far. As systemic therapy has changed only little over the years, the ensuing findings may be used as a benchmark.

The analysis does not come without limitations. For one, it is unknown which proportion of patients with UPSB cared for in contributing centers was indeed registered. As a result, it cannot be claimed that this report is truly population based. Some variables had to be investigated using somewhat incomplete datasets. However, sufficient data was available to allow analyzing even those. Of note, all patients received a recommendation for combined modality therapy. Therefore, no information on patients treated by local approaches only is available. Hence, no internal comparison to such patients is feasible. Altogether, however, the wealth of reported information seems unique and the results of the analyses well worthwhile reporting.

The series only includes tumors arising within bone. Undifferentiated pleomorphic sarcoma of the soft-tissues seems to represent a different entity altogether ( WHO Classification of Tumours 2020). There have, of course, been previous publications about UPSB, some including patients also covered by this report. One of these was an early series by the European Musculo-Skeletal Oncology Society (EMSOS). It evaluated event-free survival exclusively but did not describe overall outcomes (Bielack et al. 1999). The second described a sub-cohort of the EURO-B.O.S.S.-study, to which COSS contributed. Only patients older than 40 years at diagnosis were included (Palmerini et al. 2023). Most patients in the current series are reported for the very first time.

A detailed chart-review revealed that approximately one out of every ten tumors initially registered with COSS as UPSB was in fact not eligible for this analysis. These were tumors originally registered as UPSB but indeed representing other entities, tumors situated not in bone but in soft-tissues, and some pretreated UPSB. UPSB may not be an easy diagnosis to make (Romeo et al. 2012). The fact that almost two thirds of eligible tumors were evaluated by reference-pathology adds validity to the findings described in this report.

Remarkably, we detected few patients with documented tumor-predisposition syndromes. Osteosarcoma, on the other hand, may show detectable predispositions in around a quarter of cases (Mirabello et al. 2020). The early time-period in which some of the UPSB studied were detected makes their molecular testing unlikely. Additionally, not all positive genetic findings may have been reported. Still, the observation is of potential interest, particularly as other reports offer little information regarding this topic at all.

More than 10% of all UPSB in this series represented second primary malignancies. In these cases, treatment for the prior cancer had generally included radiotherapy. UPSB must certainly be included among tumors which may be caused by radiation.

The age at which UPSB was diagnosed in this series, a median of over 40 years, clearly distinguishes this tumor from osteosarcoma. Data from the SEER-registry point to a similar median age (Gusho et al. 2022). Osteosarcoma, particularly if located in an extremity, is strongly related to the age of maximal bone growth, adolescence. The factor of bone-growth is clearly less pronounced in UPSB. As a consequence, it is less the pediatrician but rather the medical oncologist who will face affected patients.

Other than the distribution of ages, that of genders seems to reflect that of osteosarcoma (Beird et al. 2022), males more likely affected. Again similar to osteosarcoma (Beird et al. 2022), UPSB shares a predilection for the extremities and, there, the knee, where almost three quarters of extremity UPSB were located. Some ten percent of cases affected the axial skeleton and isolated cases even craniofacial sites. It seems that any region of the body may be affected.

UPSB size at detection seems somewhat smaller than in osteosarcoma. It exceeded one third of the involved bone in less than one fifth of cases, compared to one third in the latter (Bielack et al. 2002). Despite the often limited size, however, UPSB frequently led to pathologic fractures, these affecting approximately one out of seven patients. The purely osteolytic nature is probably predisposing. Pathologic fracture usually occurred prior to tumor-diagnosis, making a high index of suspicion advisable.

Primary metastases were fairly uncommon. Those few detected favored the same sites as these do in osteosarcoma, namely the lungs and, less often, distant bones. Owing to the evolution of diagnostic techniques, some cases might have escaped detection in the earlier years covered by this study. Still, UPSB appears less likely to spread primarily than osteosarcoma.

Not surprising for a multi-center group focusing on chemotherapy, all patients in this series received systemic treatment. Regimens designed against osteosarcoma were administered (Bielack et al. 2023a). That only two thirds of UPSB-patients received pre-operative chemotherapy may be best explained by their relatively advanced age. Older individuals are known to frequently proceed straight to surgery (Ferrari et al. 2018) (Bielack et al. 2023b). The duration over which chemotherapy was administered and the drugs employed otherwise reflected standard osteosarcoma practices (Strauss et al. 2021) (Beird et al. 2022). The low frequency at which high-dose methotrexate was employed is explained by the high proportion of patients aged above 40 years. In such, methotrexate is often avoided due to concerns of toxicity (Bielack et al. 2023b).

Most UPSB in this series proceeded to surgery. For extremity tumors, limb-salvage was the preferred option. In primaries operated upon following neo-adjuvant systemic treatment, histologic tumor-response was found poor in more than sixty percent. This low response rate may for one be related age, older individuals known to exhibit poor responses more often than younger individuals (Ferrari et al. 2018; Bielack et al. 2023b). A difference in tumor biology between UPSB and osteosarcoma can also not be excluded. Too few UPSB received radiotherapy to speculate about its potential efficacy.

After treatment, more than 90% of the UPSB in this series went into complete remission. With a median follow-up period of more than five years, outcome-results may be considered sufficiently mature. Both the types of secondary malignancies occurring during this time-period and their incidence seem quite typical for what our group observed in heavily treated bone-sarcoma patients (Kube et al. 2022). The event-free survival probability, almost two thirds at five years, seems remarkable, as patients were mostly of a rather advanced age. Here, UPSB seems to behave more positively than does osteosarcoma.

If secondary metastases occurred, these generally affected the lungs and distant bones, just like osteosarcoma (Beird et al. 2022). A few individuals, however, developed recurrences at sites quite unusual for the latter tumor.

As expected, given the moderate rate of recurrences, approximately two thirds of patients survived for at least five years. Most deaths observed were directly attributable to UPSB. Some therapy related causes, secondary malignancies, unrelated causes, and unknown causes also contributed.

Several potentially relevant prognostic could be dereced. Some were expected: Patients aged less than 40 years did better than older ones. Patients with extremity primaries did better than those with tumors located elsewhere. Patients whose disease had already spread at diagnosis did worse than those with localized tumors. There were only very few affected patients in this series but their prognosis was grim. Pathologic fractures were associated with an inferior outcome. In this context, it is interesting to note that such is also found for older—but not younger—osteosarcoma patients (Kelley et al. 2020).

The reasons for ablative surgery producing better results than limb-salvage are certainly complex. Any interpretation is certainly not straightforward. Far less surprisingly, patients who failed to achieve a complete remission generally also failed to survive.

It may be interesting to take a closer look at some factors not found significant. Here, reference pathology, symptom duration, and patient gender seemed of minor impact. Remarkably, initial tumor-size did not seem predictive. This is in clear distinction to osteosarcoma (Bielack et al. 2002). Maybe even more strikingly, we could observe no significant correlation between tumor-response and prognosis, poor responders doing just as well as good responders. In osteosarcoma, on the other hand, response is one of the most important predictors of prognosis (Beird et al. 2022; Bielack et al. 2002). It seems that a poor response of an UPSB must not be a cause of unnecessary concern.

The UPSB described in this series were treated on regimens designed against osteosarcoma and not on protocols against soft-tissue sarcoma. In the latter tumors, cisplatin and methotrexate have not been proven particularly efficacious. Anthracyclines and ifosfamide, however, have very much been so. If choosing chemotherapy against UPSB, it remains open whether regimens absolutely identical to those designed against osteosarcoma represent the optimal choice. Still, in the absence of superior alternatives, these may currently represent valid options.

In summary, this series describes four decades of multi-institutional experience with UPSB treated in the German-speaking region of Europe. It depicts many clinical similarities of UPSB to osteosarcoma but also delineates some important differences. Maybe most notably, an inferior local response to chemotherapy in no way predicts systemic failures. Given the results achieved, it seems reasonable to continue choosing osteosarcoma-regimens if planning to employ chemotherapy against UPSB.

## Data Availability

The data that support the findings of this study are available from the corresponding author upon reasonable request.
